# A comparative analysis of radical cystectomy with perioperative chemotherapy, chemoradiation therapy, or systemic therapy in patients with clinically advanced node-positive bladder cancer (cN2/N3)

**DOI:** 10.3389/fonc.2023.1157880

**Published:** 2024-01-11

**Authors:** Harshit Garg, Mukund Bhandari, Furkan Dursun, Michael A. Liss, Dharam Kaushik, Robert S. Svatek, Ahmed M. Mansour

**Affiliations:** ^1^ Department of Urology, University of Texas Health, San Antonio, TX, United States; ^2^ Department of Population Health Science, University of Texas Health, San Antonio, TX, United States; ^3^ Department of Urology, University of Texas Health San Antonio/MD Anderson Mays Cancer Center, San Antonio, TX, United States; ^4^ Urology and Nephrology Center, Mansoura University, Mansoura, Egypt

**Keywords:** locally advanced bladder cancer, radical cystectomy, concurrent chemoradiation, bladder cancer, overall survival

## Abstract

**Introduction:**

The management of non-metastatic clinically advanced lymph nodal (cN2/N3) bladder cancer (Stage IIIB) could involve radical cystectomy, chemoradiation, or systemic therapy alone. However, a definitive comparison between these approaches is lacking. This study aims to compare the outcomes of patients undergoing radical cystectomy with pelvic lymph node dissection (RC-PLND), chemoradiation therapy (CRT) or systemic therapy (including immunotherapy) (ST) only in patients with stage IIIB bladder cancer.

**Materials and methods:**

A retrospective analysis of the National Cancer Database for patients with stage IIIB urothelial bladder cancer was done from 2004-2019. Patients were classified as Group A: Those who received RC-PLND with perioperative chemotherapy, Group B: Those who received CRT, and Group C: Those who received only ST alone. The primary outcome was overall survival (OS). Inverse probability weighting (IPW)-adjusted Kaplan Meier curves were utilized to compare overall survival (OS) and cox multivariate regression analysis was used to identify predictors for OS.

**Results:**

Overall, 2,575 patients were identified. They were classified into Group A (n=1,278), Group B (n=317) and Group C (n=980). Compared to Group B, patients in Group A were younger (SMD=19.6%), had lower comorbidities (SMD=18.2%), had higher income (SMD=31.5%), had private insurance (SMD= 26.7%), were treated at academic centres (SMD=29.3%) and had higher percentage of N2 disease (SMD=31.1%). Using IPW-adjusted survival analysis, compared to Group C, the median OS was significantly higher in Group A (20.7 vs 14.2 months, p<0.001) and Group B (19.7 vs 14.2 months, p<0.001) but similar between Group A and Group B (20.9 vs 19.7 months, p=0.74). Both surgery (HR=0.72 (0.65-0.80), p<0.001) and CRT (0.70 (0.59-0.82), p<0.001) appeared to be independent predictors for OS on cox-regression analysis. The major limitations include bias due to retrospective analysis and non-assessment of cancer-specific survival.

**Conclusion:**

In stage IIIB bladder cancer with advanced lymph nodal disease, both RC and CRT offer equivalent survival benefits and are superior to systemic therapy alone.

## 1 Introduction

Neoadjuvant chemotherapy followed by radical cystectomy with pelvic lymph node dissection (RC-PLND) is the standard management of non-metastatic muscle-invasive bladder cancer (1). However, the management of clinically node-positive bladder cancer remains controversial. Although potentially curable, such patients have a high risk for distant metastasis. They were associated with a dismal prognosis and were included in stage IV bladder cancer as per the 7^th^ edition American Joint Committee on Cancer (AJCC) staging (2). Most of the major clinical trials on radical cystectomy excluded patients with lymph node-positive disease (3, 4). Systemic therapy (ST) or chemoradiotherapy was considered the standard of care (5). However, with the changes in the recent 8^th^ AJCC staging (6), node-positive bladder cancer is included under stage III with N1 disease (single regional lymph node metastasis in the true pelvis) sub-stratified as stage IIIA and N2 (multiple regional lymph node metastases in the true pelvis) or N3 disease (lymph node metastasis to common iliac lymph nodes) as stage IIIB. As per the recent National Comprehensive Cancer Network (NCCN) (v 2.2022) guidelines (1), the management of stage IIIA bladder cancer involves neoadjuvant cisplatin-based combination chemotherapy followed by RC-PLND or bladder-preservation multimodality treatment. However, the management of non-metastatic clinically advanced lymph nodal disease (stage IIIB) disease remains debatable and includes either downstaging systemic therapy followed by RC-PLND or concurrent chemoradiotherapy (CRT). Though few studies have compared CRT or RC-PLND with ST in node-positive bladder cancer (7–9), a definitive comparison between RC-PLND, CRT, and ST in advanced node-positive bladder cancer is lacking. Furthermore, with limited numbers of non-metastatic N2/N3 disease patients, most comparative studies include a large proportion of N1 disease patients, which introduces an inherent bias in favor of radical treatment, thereby, making definitive inference difficult. As the experience with RC-PLND and CRT has increased and treatment of the disease in non-metastatic settings still provides an opportunity for a definitive cure, it becomes imperative to assess their role in locally advanced (cN2/N3) bladder cancer. For this, we conducted a comparative analysis between RC, CRT, and systemic therapy using a contemporary dataset of the National Cancer Database (NCDB) on patients with stage IIIB bladder cancer.

## 2 Materials and methods

### 2.1 Source of data

The NCDB is jointly sponsored by the American College of Surgeons and the American Cancer Society, which is sourced from hospital registry data that are collected in more than 1,500 Commission on Cancer (CoC)-accredited facilities. This database currently captures >70% of all newly diagnosed cancer cases, and currently contains approximately 34 million individual patient records from hospital cancer registries across the United States (10). Collected data include patient demographic, tumor, facility, and treatment characteristics. This study is exempt from Institutional Review Board Approval as there is no identifiable patient information in the NCDB.

### 2.2 Study population

**Figure 1:**
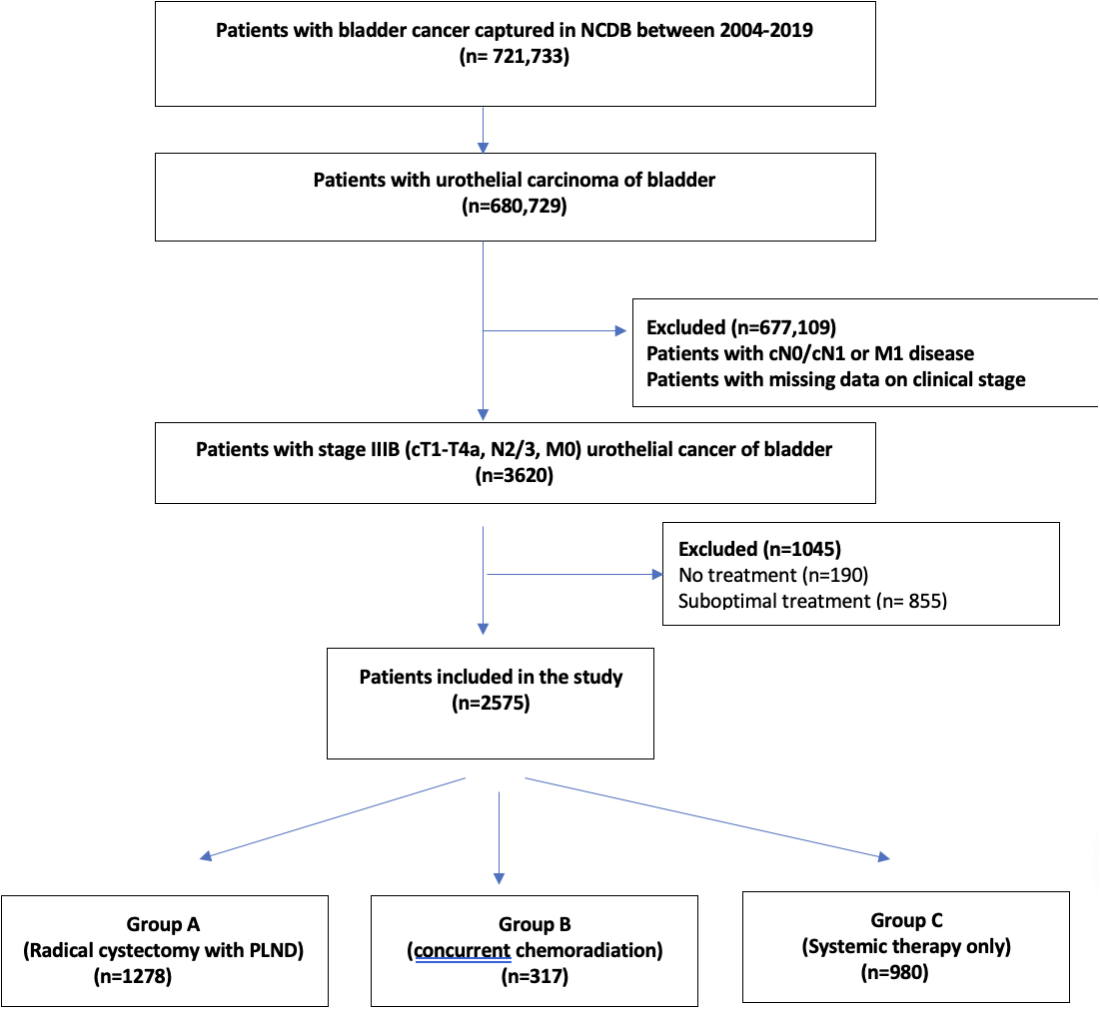
Flowchart depicting the selection of stage IIIB urinary bladder cancer patients, who received radical cystectomy with pelvic lymph node dissection or concurrent chemoradiation therapy or systemic therapy alone.

A total of 721,733 patients who have been diagnosed with bladder cancer between 2004 and 2019 in the NCDB (International Classification of Diseases for Oncology, 3rd edition topography codes C67.0- C67.9) have been investigated. We further identified patients with urothelial bladder cancer with stage cT1-T4aN2-3M0 (6). Patients were then classified as Group A: Those who received definitive surgery i.e. RC-PLND with neoadjuvant/adjuvant therapy; Group B: Those who received CRT (defined as the use of ≥ 60Gray of radiation therapy with single or multiagent chemotherapy after transurethral resection of bladder tumor); Group C: Patients who received ST alone. **Supplementary Appendix** provides codes used for data extraction from NCDB (**Figure 1**).

### 2.3 Covariates and endpoints

In addition to treatment modalities, studied parameters included clinical variables as clinical T-stage and nodal stage (N-stage), sociodemographic variables such as gender, age, race, ethnicity, Charlson-Deyo comorbidity index (CCI), median income, insurance status, level of education, urbanization, and facility type for treatment.

### 2.4 Outcome measure

The primary endpoint was the overall survival (OS) in each cohort, defined as the time from cancer diagnosis to death or the date of the last follow-up as recorded in the NCDB.

### 2.5 Statistical analysis

Descriptive statistics were reported using medians and interquartile ranges for continuous variables, and frequencies and proportions for categorical variables. The standardized difference approach was used to compare covariates among different treatment modalities for assessment of possible confounding. A standardized mean difference (SMD) of >10% for a given covariate indicated a significant imbalance (11). Kaplan-Meier analysis was used to compare survival between the three groups. Cox proportional hazard modeling was fitted to identify demographic, clinical, and treatment variables associated with survival. To account for selection bias, observed differences in baseline characteristics between patients of the three groups were controlled using inverse probability weighting (IPW) propensity score analysis. Propensity scores were generated on a multivariate regression model using risk groups as the dependent variable. The covariates balanced included age, CCI, race, ethnicity, insurance, education, income, facility type, clinical T stage, and clinical N stage. Inclusion p-values were 0.05. IPW-adjusted Kaplan-Meier curves were used to compare OS between the three groups. To assess independent predictors of OS, an IPW-adjusted Cox proportional hazards regression model was fitted to account for confounders (12). Bonferroni correction method was used to counteract the false positive results from multiple comparisons. For missing data, the omission method was utilized as less than 1% of the data used in analyses was missing. All statistical analysis was performed using R (version 4.2.1) software with a two-sided p-value of 0.05 determined as being statically significant.

## 3 Results

### 3.1 Patient and treatment characteristics

Of 721,733 patients with urinary bladder cancer captured in NCDB between 2004-2019, 2575 patients with non-metastatic bladder cancer with cT1-4aN2/3M0 (stage IIIB) bladder cancer were included in the study (**Figure 1**). Amongst them, 1278 patients were included in group A (RC-PLND), 317 patients were included in group B (CRT) and 980 patients were included in group C (ST).

The mean age (SD) of the study population was 65.7(10.6) years and 73% (1880/2575) of patients were males. **Tables 1**–**3** highlights the demographic and clinicopathological characteristics of the study cohort.

**Table 1:** Demographics and clinical characteristics of patients with stage IIIB bladder cancer undergoing radical cystectomy and pelvic lymph node dissection (Group A) and concurrent chemoradiation therapy (Group B) in unweighted population and weighted study population.

Parameter	Unweighted population			Weighted population		
	Group A	Group B	Standardized difference (%)	Group A	Group B	Standardized difference (%)
**Number of patients**	1278	317		1333	1356	
**Mean age (SD), years**	64.9 (10.6)	66.9 (10.3)	19.6			
**Age, n(%)**			14.6			7.7
<60	382 (29.9)	78 (24.6)		335(25.1)	358 (26.4)	
60 - 69	437 (34.2)	112 (35.3)		505 (37.9)	486 (35.9)	
70 - 79	354 (27.7)	91 (28.7)		353 (26.5)	390 (28.8)	
≥80	105 (8.2)	36 (11.4)		140 (10.5)	122 (9.0)	
**Gender**			11			
Female	324 (25.4)	96 (30.3)				
Male	954 (74.6)	221 (69.7)				
**Race, n(%)**			11.8			5.8
White	1143 (89.4)	273 (86.1)		1202(88.7)	1202.4 (88.7)	
Black	98 (7.7)	34 (10.7)		130 (9.8)	111.1 (8.2)	
Asian	18 (1.4)	4 (1.3)		22 (1.6)	19.4 (1.4)	
Others	9 (0.7)	2 (0.6)		10 (0.8)	11 (0.8)	
Unknown	10 (0.8)	4 (1.3)		12 (0.9)	12 (0.9)	
**Ethnicity, n(%)**			8.5			3.3
Non-Hispanic	1173 (91.8)	293 (92.4)		1211.2 (90.9)	1244.7 (91.8)	
Hispanic	46 (3.6)	14 (4.4)		57.4 (4.3)	51.7 (3.8)	
Unknown	59 (4.6)	10 (3.2)		64.2 (4.8)	59.5 (4.4)	
**Charlson Comorbidity Index (CCI)**			18.2			6.8
CCI=0	928 (72.6)	221 (69.7)	18.2	955.7 (71.7)	972.0 (71.7)	
CCI=1	252 (19.7)	58 (18.3)		239.0 (17.9)	264.8 (19.5)	
CCI=2	69 (5.4)	20 (6.3)		97.9 (7.3)	79.7 (5.9)	
CCI=3	29 (2.3)	18 (5.7)		40.3 (3.0)	39.5 (2.9)	
**Urbanization**			9.3			0.3
Rural	24 (2.0)	10 (3.3)		26.8 (2.0)	27.8 (2.1)	
Urban	198 (16.2)	44 (14.4)		211.7 (15.9)	215.6 (15.9)	
Metro Areas	1001 (81.8)	252 (82.4)		1094.3 (82.1)	1112.5 (82.0)	
**Insurance Type**			26.7			10
Not Insured	40 (3.1)	15 (4.7)		44.3 (3.3)	42.2 (3.1)	
Private Insurance	440 (34.4)	83 (26.2)		402.0 (30.2)	442.2 (32.6)	
Medicaid	97 (7.6)	27 (8.5)		98.7 (7.4)	94.1 (6.9)	
Medicare	663 (51.9)	188 (59.3)		773.5 (58.0)	739.0 (54.5)	
Other Government	19 (1.5)	4 (1.3)		14.4 (1.1)	19.4 (1.4)	
Unknown	19 (1.5)	0 (0.0)		0.0 (0.0)	19.0 (1.4)	
**Education**			17.7			4.1
≥21%	191 (16.6)	44 (15.5)		238.0 (17.9)	224.6 (16.6)	
13-20.9%	301 (26.2)	85 (29.9)		358.8 (26.9)	359.6 (26.5)	
7%-12.9%	379 (33.0)	105 (37.0)		439.1 (32.9)	457.0 (33.7)	
<7%	278 (24.2)	50 (17.6)		296.9 (22.3)	314.7 (23.2)	
**Income**			31.5			10.0
< $38,000	191 (16.6)	51 (18.0)		265.2 (19.9)	229.6 (16.9)	
$38,000 - $47,999	277 (24.1)	74 (26.1)		301.0 (22.6)	322.1 (23.8)	
$48,000 - $62,999	296 (25.8)	101 (35.6)		414.7 (31.1)	380.0 (28.0)	
>=$63,000	384 (33.4)	58 (20.4)		352.0 (26.4)	424.2 (31.3)	
**Facility Type**			29.3			5.1
Academic/Research Program	678 (53.9)	127 (40.4)		689.5 (51.7)	677.7 (50.0)	
Community Cancer Program	60 (4.8)	23 (7.3)		70.9 (5.3)	77.3 (5.7)	
Comprehensive Community Cancer Program	329 (26.2)	115 (36.6)		390.8 (29.3)	394.6 (29.1)	
Integrated Network	190 (15.1)	49 (15.6)		181.7 (13.6)	206.2 (15.2)	
**Preoperative cT stage**			45.1			4.1
cT1	66 (5.2)	35 (11.0)		75.6 (5.7)	79.6 (5.9)	
cT2	501 (39.2)	173 (54.6)		529.7 (39.7)	560.2 (41.3)	
cT3	386 (30.2)	61 (19.2)		409.6 (30.7)	388.8 (28.7)	
cT4	325 (25.4)	48 (15.1)		317.9 (23.9)	327.3 (24.1)	
**cN stage**			31.1			1.1
cN2, n(%)	1078 (84.4)	227 (71.6)		1103.5 (82.8)	1114.8 (82.2)	
cN3, n(%)	200 (15.6)	90 (28.4)		229.4 (17.2)	241.2 (17.8)	

**Table 2:** Demographics and clinical characteristics of patients with stage IIIB bladder cancer undergoing radical cystectomy and pelvic lymph node dissection (Group A) vs systemic therapy alone (Group C) in an unweighted population and weighted study population.

Parameter	Unweighted population			Weighted population		
	Group A	Group C	Standardized difference (%)	Group A	Group C	Standardized difference (%)
**Number of patients**	1278	980		1949.6	1919.4	
**Mean age (SD), years**	64.9 (10.6)	66.50 (10.70)	15.2			
**Age, n(%)**			14.5			1.5
<60	382 (29.9)	263 (26.8)		524.4 (26.9)	515.9 (26.9)	
60 - 69	437 (34.2)	318 (32.4)		667.6 (34.2)	657.9 (34.3)	
70 - 79	354 (27.7)	278 (28.4)		556.6 (28.5)	555.4 (28.9)	
≥80	105 (8.2)	121 (12.3)		201.0 (10.3)	190.2 (9.9)	
**Gender**			6.1			1.0
Female	324 (25.4)	275 (28.1)		508.7 (26.1)	511.8 (26.7)	
Male	954 (74.6)	705 (71.9)		1440.9 (73.9)	1407.6 (73.3)	
**Race, n(%)**						1.1
White	1143 (89.4)	872 (89.0)	6.5	1730.1 (88.7)	1704.2 (88.8)	
Black	98 (7.7)	85 (8.7)		159.9 (8.2)	155.5 (8.1)	
Asian	18 (1.4)	9 (0.9)		27.0 (1.4)	25.6 (1.3)	
Others	9 (0.7)	5 (0.5)		13.4 (0.7)	14.5 (0.8)	
Unknown	10 (0.8)	9 (0.9)		19.2 (1.0)	19.7 (1.0)	
**Ethnicity, n(%)**			4.9			0.2
Non-Hispanic	1173 (91.8)	887 (90.5)		1779.9 (91.3)	1751.9 (91.3)	
Hispanic	46 (3.6)	44 (4.5)		76.0 (3.9)	75.6 (3.9)	
Unknown	59 (4.6)	49 (5.0)		93.7 (4.8)	92.0 (4.8)	
**Charslon Co-morbidity Index**			7.8			
						0.7
CCI=0	928 (72.6)	692 (70.6)	7.8	1398.8 (71.7)	1378.2 (71.8)	
CCI=1	252 (19.7)	194 (19.8)		384.0 (19.7)	380.2 (19.8)	
CCI=2	69 (5.4)	61 (6.2)		116.1 (6.0)	112.3 (5.9)	
CCI=3	29 (2.3)	33 (3.4)		50.7 (2.6)	48.6 (2.5)	
**Urbanization**			2.7			1.1
Rural	24 (2.0)	22 (2.3)		37.5 (1.9)	39.2 (2.0)	
Urban	198 (16.2)	149 (15.8)		302.0 (15.5)	301.6 (15.7)	
Metro Areas	1001 (81.8)	771 (81.8)		1610.1 (82.6)	1578.6 (82.2)	
**Insurance Type**			11.1			1.3
Not Insured	40 (3.1)	41 (4.2)		70.6 (3.6)	66.2 (3.4)	
Private Insurance	440 (34.4)	300 (30.6)		627.7 (32.2)	619.0 (32.3)	
Medicaid	97 (7.6)	83 (8.5)		140.5 (7.2)	137.2 (7.2)	
Medicare	663 (51.9)	524 (53.5)		1048.9 (53.8)	1035.5 (53.9)	
Other Government	19 (1.5)	11 (1.1)		23.0 (1.2)	24.3 (1.3)	
Unknown	19 (1.5)	21 (2.1)		39.0 (2.0)	37.2 (1.9)	
**Education**			7.5			1.2
≥21%	191 (16.6)	133 (15.0)		305.7 (15.7)	307.9 (16.0)	
13-20.9%	301 (26.2)	238 (26.9)		513.8 (26.4)	500.6 (26.1)	
7%-12.9%	379 (33.0)	316 (35.7)		660.9 (33.9)	653.5 (34.0)	
<7%	278 (24.2)	197 (22.3)		469.2 (24.1)	457.4 (23.8)	
**Income**			9.7			0.6
< $38,000	191 (16.6)	157 (17.8)		333.9 (17.1)	326.3 (17.0)	
$38,000 - $47,999	277 (24.1)	210 (23.8)		460.9 (23.6)	450.6 (23.5)	
$48,000 - $62,999	296 (25.8)	256 (29.0)		533.1 (27.3)	525.7 (27.4)	
>=$63,000	384 (33.4)	261 (29.5)		621.7 (31.9)	616.8 (32.1)	
**Facility Type**			24.8			2.0
Academic/Research Program	678 (53.9)	407 (41.8)		937.6 (48.1)	916.8 (47.8)	
Community Cancer Program	60 (4.8)	65 (6.7)		111.6 (5.7)	117.7 (6.1)	
Comprehensive Community Cancer Program	329 (26.2)	327 (33.6)		592.2 (30.4)	576.4 (30.0)	
Integrated Network	190 (15.1)	175 (18.0)		308.1 (15.8)	308.5 (16.1)	
**Preoperative cT stage**			42.3			1.7
cT1	66 (5.2)	125 (12.8)		155.9 (8.0)	157.2 (8.2)	
cT2	501 (39.2)	490 (50.0)		818.5 (42.0)	815.6 (42.5)	
cT3	386 (30.2)	173 (17.7)		511.1 (26.2)	490.1 (25.5)	
cT4	325 (25.4)	192 (19.6)		464.1 (23.8)	456.5 (23.8)	
**cN stage**			22.4			0.4
cN2, n(%)	1078 (84.4)	739 (75.4)		1584.5 (81.3)	1556.6 (81.1)	
cN3, n(%)	200 (15.6)	241 (24.6)		365.1 (18.7)	362.8 (18.9)	

**Table 3:** Demographics and clinical characteristics of patients with stage IIIB bladder cancer concurrent chemoradiation therapy (Group B) vs systemic therapy alone (Group C) in unweighted population and weighted study population.

Parameter	Unweighted population			Weighted population		
	Group B	Group C	Standardized difference (%)	Group B	Group C	Standardized difference (%)
**Number of patients**	317	980		1115.5	1089.5	
**Mean age (SD), years**	66.9 (10.3)	66.50 (10.70)	4.1			
**Age, n(%)**			7.3			1.7
<60	78 (24.6)	263 (26.8)		276.9 (24.8)	264.0 (24.2)	
60 - 69	112 (35.3)	318 (32.4)		379.8 (34.0)	373.8 (34.3)	
70 - 79	91 (28.7)	278 (28.4)		315.7 (28.3)	307.7 (28.2)	
≥80	36 (11.4)	121 (12.3)		143.1 (12.8)	143.9 (13.2)	
**Gender**			6.1			2.8
Female	96 (30.3)	275 (28.1)		323.3 (29.0)	329.9 (30.3)	
Male	221 (69.7)	705 (71.9)		792.1 (71.0)	759.6 (69.7)	
**Race, n(%)**			8.8			1.0
White	273 (86.1)	872 (89.0)		982.7 (88.1)	958.6 (88.0)	
Black	34 (10.7)	85 (8.7)		100.9 (9.0)	99.8 (9.2)	
Asian	4 (1.3)	9 (0.9)		12.1 (1.1)	12.3 (1.1)	
Others	2 (0.6)	5 (0.5)		6.8 (0.6)	6.0 (0.6)	
Unknown	4 (1.3)	9 (0.9)		12.9 (1.2)	12.7 (1.2)	
**Ethnicity, n(%)**			9.4			1.5
Non-Hispanic	293 (92.4)	887 (90.5)		1013.2 (90.8)	991.7 (91.0)	
Hispanic	14 (4.4)	44 (4.5)		50.2 (4.5)	50.2 (4.6)	
Unknown	10 (3.2)	49 (5.0)		52.1 (4.7)	47.5 (4.4)	
**Charslon Co-morbidity Index**			11.5			1.1
CCI=0	221 (69.7)	692 (70.6)		770.2 (69.0)	753.2 (69.1)	
CCI=1	58 (18.3)	194 (19.8)		233.1 (20.9)	224.4 (20.6)	
CCI=2	20 (6.3)	61 (6.2)		70.2 (6.3)	71.1 (6.5)	
CCI=3	18 (5.7)	33 (3.4)		42.0 (3.8)	40.7 (3.7)	
**Urbanization**			6.7			1.0
Rural	10 (3.3)	22 (2.3)		25.8 (2.3)	24.1 (2.2)	
Urban	44 (14.4)	149 (15.8)		163.3 (14.6)	162.5 (14.9)	
Metro Areas	252 (82.4)	771 (81.8)		926.4 (83.0)	902.9 (82.9)	
**Insurance Type**			24.0			9.1
Not Insured	15 (4.7)	41 (4.2)		46.9 (4.2)	45.1 (4.1)	
Private Insurance	83 (26.2)	300 (30.6)		327.4 (29.4)	317.0 (29.1)	
Medicaid	27 (8.5)	83 (8.5)		84.4 (7.6)	83.6 (7.7)	
Medicare	188 (59.3)	524 (53.5)		623.7 (55.9)	629.6 (57.8)	
Other Government	4 (1.3)	11 (1.1)		14.1 (1.3)	14.3 (1.3)	
Unknown	0 (0.0)	21 (2.1)		19.0 (1.7)	0.0 (0.0)	
**Education**			12.1			2.2
≥21%	44 (15.5)	133 (15.0)		171.1 (15.3)	175.8 (16.1)	
13-20.9%	85 (29.9)	238 (26.9)		307.7 (27.6)	299.3 (27.5)	
7%-12.9%	105 (37.0)	316 (35.7)		403.8 (36.2)	389.3 (35.7)	
<7%	50 (17.6)	197 (22.3)		232.8 (20.9)	225.1 (20.7)	
**Income**			22.2			2.7
< $38,000	51 (18.0)	157 (17.8)		191.3 (17.1)	195.1 (17.9)	
$38,000 - $47,999	74 (26.1)	210 (23.8)		274.0 (24.6)	261.7 (24.0)	
$48,000 - $62,999	101 (35.6)	256 (29.0)		343.7 (30.8)	341.5 (31.3)	
>=$63,000	58 (20.4)	261 (29.5)		306.5 (27.5)	291.2 (26.7)	
**Facility Type**			8.4			1.5
Academic/Research Program	127 (40.4)	407 (41.8)		444.3 (39.8)	434.9 (39.9)	
Community Cancer Program	23 (7.3)	65 (6.7)		80.3 (7.2)	78.4 (7.2)	
Comprehensive Community Cancer Program	115 (36.6)	327 (33.6)		406.2 (36.4)	401.5 (36.9)	
Integrated Network	49 (15.6)	175 (18.0)		184.7 (16.6)	174.7 (16.0)	
**cT stage**			13.9			2.9
cT1	35 (11.0)	125 (12.8)		131.7 (11.8)	124.3 (11.4)	
cT2	173 (54.6)	490 (50.0)		569.6 (51.1)	565.5 (51.9)	
cT3	61 (19.2)	173 (17.7)		202.3 (18.1)	202.6 (18.6)	
cT4	48 (15.1)	192 (19.6)		211.8 (19.0)	197.1 (18.1)	
**cN stage**			8.6			0.01
cN2, n(%)	227 (71.6)	739 (75.4)		834.8 (74.8)	814.5 (74.8)	
cN3, n(%)	90 (28.4)	241 (24.6)		280.7 (25.2)	274.9 (25.2)	

On comparison of patients undergoing surgery (Group A) with CRT (Group B), patients undergoing surgery were significantly younger (64.9 years vs 66.9years, SMD=19.6%), had lower comorbidities (CCI**≥**2: 7.7% vs 12%, SMD=18.2%) had higher education (SMD=17.7%), income (SMD=31.5%) and private insurance (34.4% vs 26.2%, SMD=26.7%), and were treated more frequently at academic/research programs (53.9% vs 40.4%, SMD=29.3%). Furthermore, patients undergoing surgery (Group A) had significantly higher cT3/4 stage and lower cN3 stage as compared to patients undergoing CRT (Group B) or ST (Group C) (**Tables 1**–**3**).

### 3.2 Survival analysis

**Figure 2:**
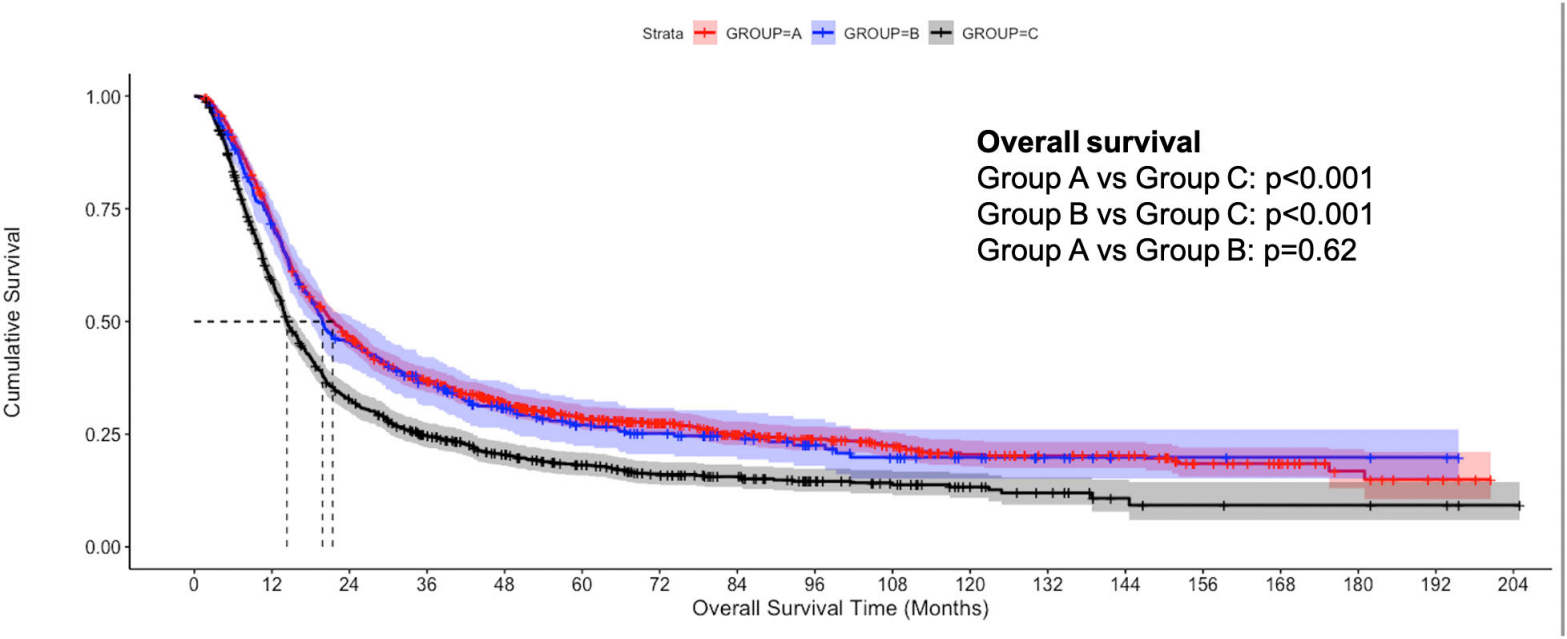
Unadjusted Kaplan-Meier estimates of overall survival in patients with stage IIIB bladder cancer undergoing radical cystectomy with pelvic lymph node dissection (Group A), concurrent chemoradiation therapy (Group B), and systemic therapy (Group C).

**Figure 3:**
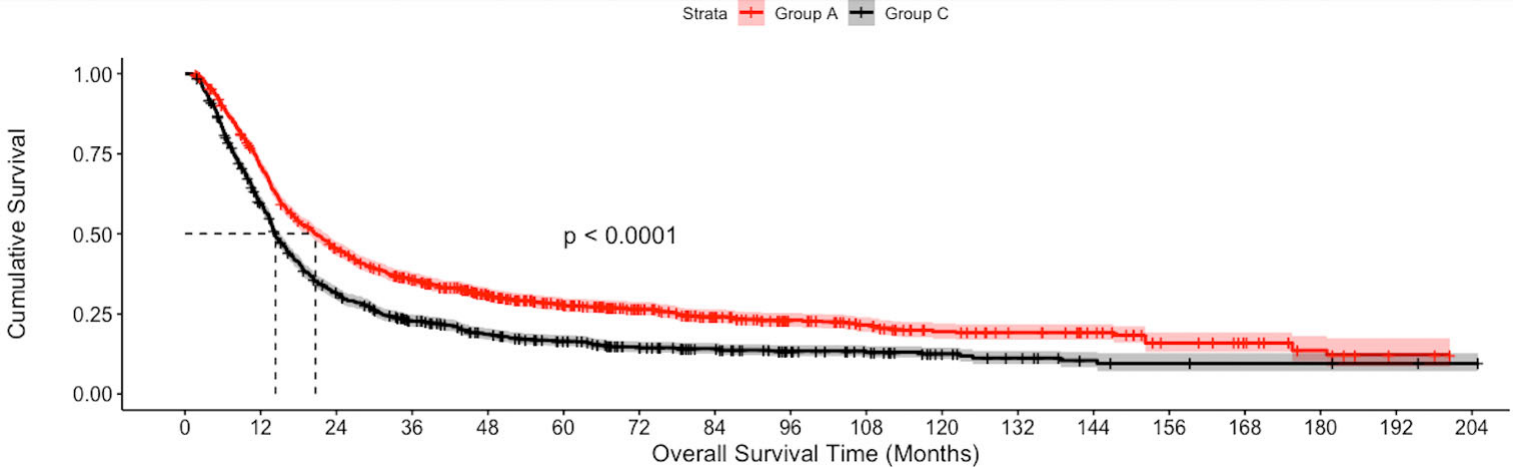
Inverse probability weighted Kaplan-Meier estimates of overall survival in patients with stage IIIB bladder cancer undergoing Radical cystectomy with pelvic lymph node dissection (Group A) vs systemic therapy only (Group C).

**Figure 4:**
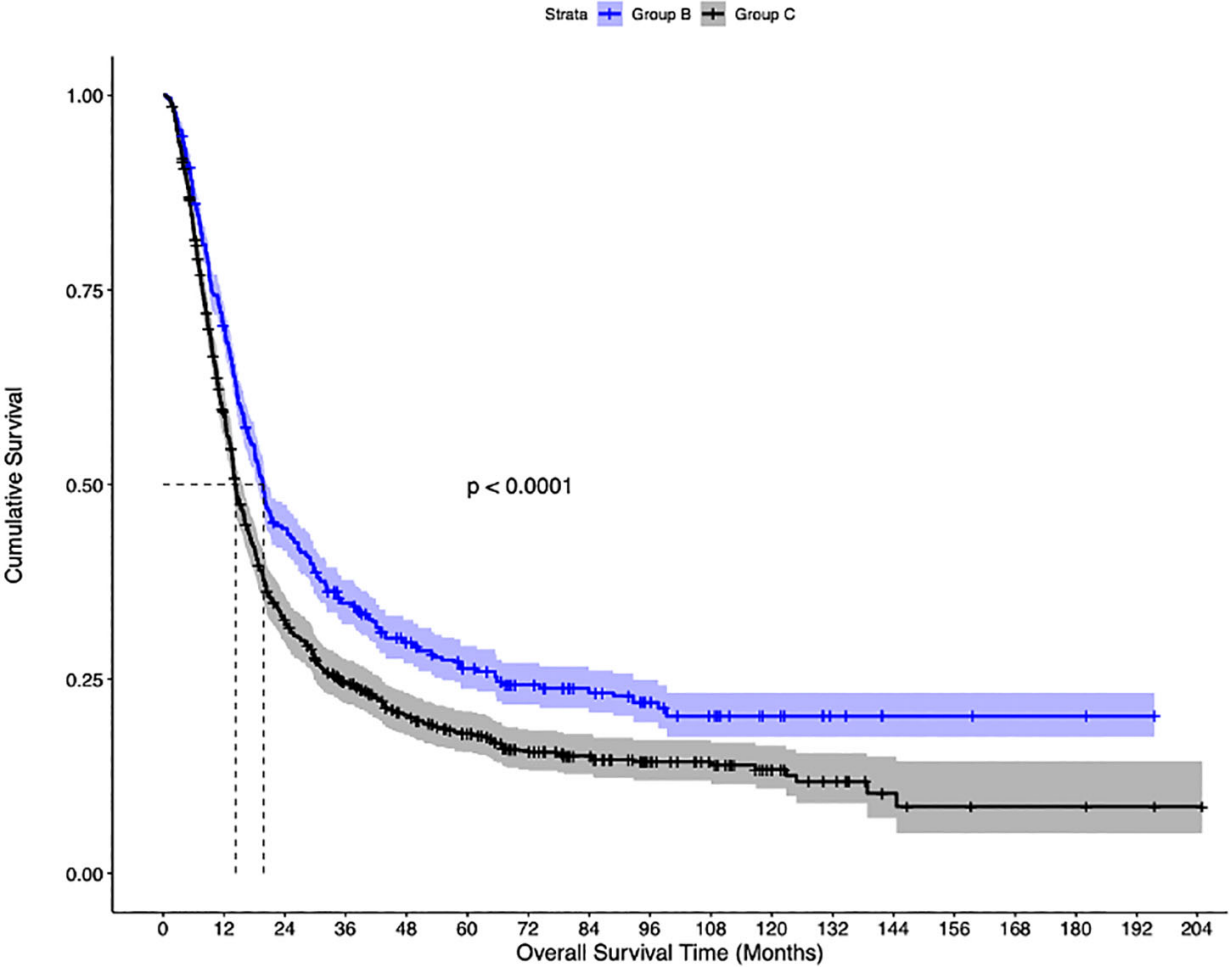
Inverse probability-weighted Kaplan-Meier estimates of overall survival in patients with stage IIIB bladder cancer undergoing concurrent chemoradiation therapy (Group B), vs systemic therapy (Group C).

**Figure 5:**
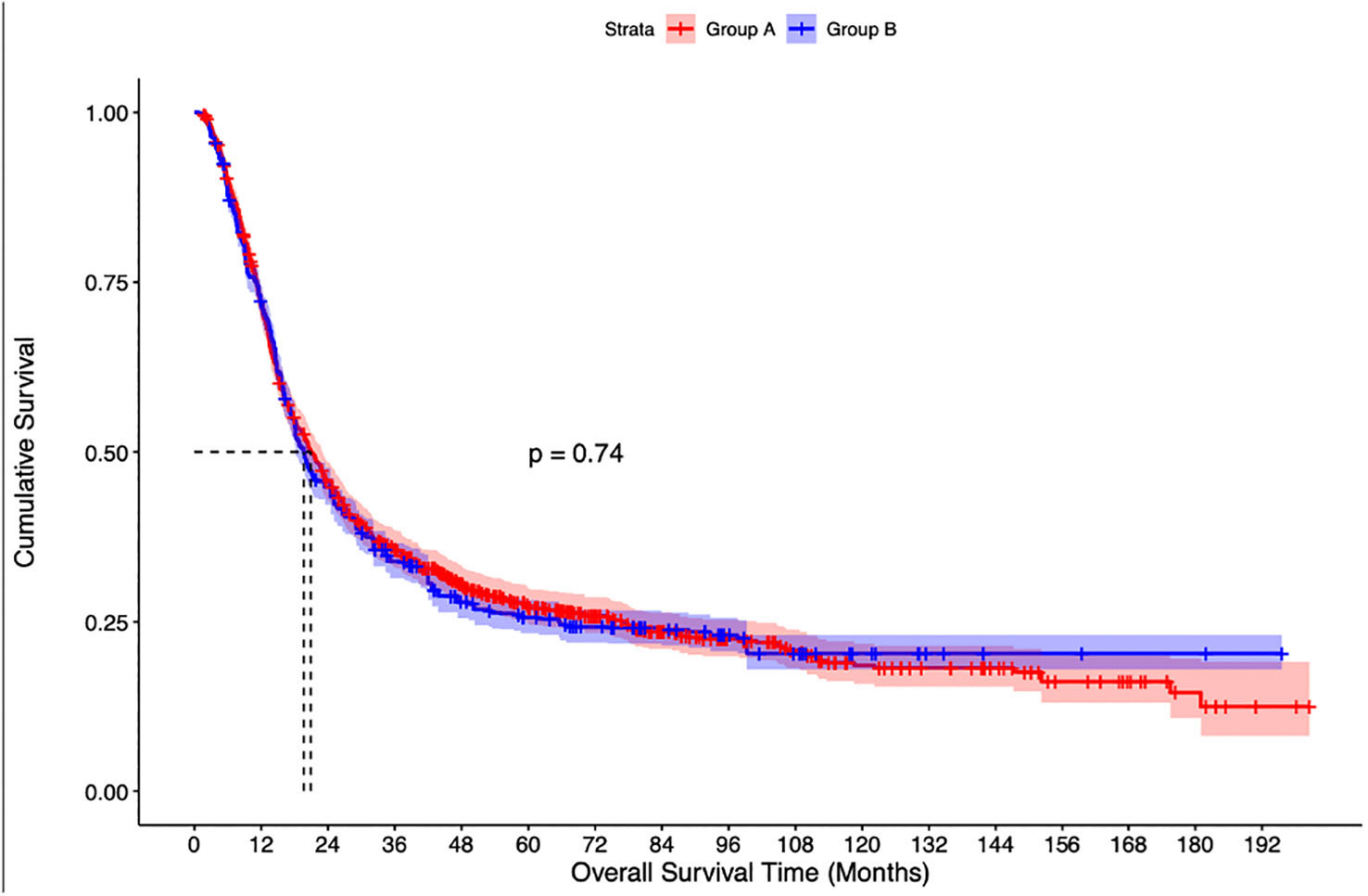
Inverse probability weighted Kaplan-Meier estimates of overall survival in patients with stage IIIB bladder cancer undergoing radical cystectomy with pelvic lymph node dissection (Group A) vs Concurrent chemoradiation therapy (Group B).

The median follow-up of the overall cohort was 17.5(9.5-43.1) months. As compared to Group C, the unadjusted median OS was significantly higher in Group A [21.4 months vs 14.3 months, p<0.001] and Group B [19.8 months vs 14.3 months, p<0.001] (**Figure 2**). Using IPW-adjusted Kaplan Meier curve analysis, the survival difference remained statistically different for Group A vs Group C [20.7 vs 14.2 months, p<0.001] (**Figure 3**) and Group B vs Group C [19.7 vs 14.2 months, p<0.001] (**Figure 4**) respectively. However, the unadjusted and IPW-adjusted median OS was similar in both Group A and Group B [21.4 vs 19.8 months, p=0.62 and 20.9 vs 19.7 months, p=0.74) (**Figures 2**, **5**).

### 3.3 Predictors for overall survival

Both RC-PLND (HR=0.72, p<0.001) and concurrent CRT (HR=0.70, p<0.001) appeared as independent predictors for OS on multivariate regression analysis. Using IPW-adjusted cox regression model for RC-PLND vs CRT, age more than 80 years (HR=1.49, p<0.001), male gender (HR=1.35, p=0.018), Hispanic ethnicity (HR= 0.47, p<0.001), higher comorbidities (HR=1.45, p=0.006), income greater than **≥** 63,000(HR=0.64, p<0.001), cT4 stage (HR=1.78, p<0.001) were independent predictors for OS. (**Supplementary Table 1**). Furthermore, in patients undergoing RC-PLND, lymph node dissection involving the removal of 16 or higher lymph nodes was an independent predictor of OS [HR= 0.85(95%CI,0.73-0.98), p=0.026].

## 4 Discussion

Management of locally advanced bladder cancer with cN2/N3 disease has remained a challenge. As experience with definitive therapies such as RC-PLND and CRT is increasing, their role needs better elucidation for a possible definitive cure of this patient population. In our study, we found both RC and CRT associated with superior OS as compared to systemic therapy alone, even after propensity score weighted analysis. Furthermore, both RC-PLND and CRT were independent predictors for overall survival.

Lymphatic vessels have been referred to as ‘highways’ for tumor metastases and the crosstalk between the primary tumor and the lymphatics plays a key role in priming the premetastatic niche (13, 14). Hence, controlling the loco-regional tumor might have a potential role in the control of the disease. The lymph node involvement in bladder cancer has been reported in approximately 30% of cases with cT2 disease and 60% with cT3 or greater disease (15). Previously, nodal involvement in bladder cancer was staged together with metastatic disease (2), thereby, implying a limited role of local therapy. However, with recent changes in AJCC staging (6) and NCCN guidelines (1), radical treatment is currently recommended for cN1 disease (stage IIIA), however, evidence for radical treatment in support of stage IIIB disease remains limited. Such debate extends into other genitourinary cancers with advanced lymph node disease. The role of definitive therapy such as radical surgery or radiation therapy has been a point of contemplation, depending on the life expectancy of the patient and the malignancy type. For instance, Seisen et al. (16) showed a survival benefit of radical treatment i.e. radical prostatectomy or radiation therapy as associated with a survival benefit compared to androgen deprivation therapy alone in prostate cancer, while in upper tract urothelial cancer, the nodal disease is staged with metastatic disease as stage IV (6) and role of radical treatment remains unclear (17).

Few studies have tried to assess various treatment modalities in node-positive bladder cancer.

Stokes et al. (18) studied the role of definitive RT (≥54 Gy) in 392 patients with node-positive bladder cancer and reported a significant overall survival benefit with the use of RT, irrespective of receipt of chemotherapy. Tan et al. (19) further showed the feasibility of intensity-modulated radiotherapy to pelvic nodes and bladder, with or without chemotherapy, for successful regional control in 38 patients with node-positive bladder cancer, with low rates of genitourinary and gastrointestinal toxicity and a 5-year OS of 34%. In another study, Haque et al. (7) compared CRT (defined as dose >55Gy) with chemotherapy alone for node-positive (N1-3) bladder cancer patients using NCDB from 2004-2013. They reported higher median OS with CRT as compared to systemic therapy alone (19 months vs 13.8 months) and the use of CRT was an independent predictor of survival. In this study, 44.5% of patients had cN1 disease and only 22.2% of the entire cohort received CRT. Similarly, few studies have compared surgery with systemic therapy in node-positive bladder cancer. Galsky et al. (9) analyzed 1739 patients with cN1-3 bladder cancer patients. Amongst them, 1104 patients undergoing RC with or without neoadjuvant chemotherapy were compared with 635 patients treated with chemotherapy alone. The 5-year OS for chemotherapy alone was significantly lower than for definitive surgery (14% vs 19%). Furthermore, neoadjuvant systemic therapy offered superior survival benefits as compared to adjuvant systemic therapy. However, they did not attempt to compare the role of CRT and their analysis was not matched to address the potential confounding factors.

A recent study by Sood et al. attempted to address the role of high-intensity local treatment with conservative treatment in node-positive bladder cancer using the NCDB (8). They compared 784 patients undergoing high-intensity local treatment (RC-PLND or CRT) with 2,443 patients managed conservatively. They reported significantly superior 5-year OS with local treatment (28.4%) as compared to conservative treatment (18.3%). Furthermore, they compared RC-PLND with CRT and found a higher 5-year OS with RC-PLND (31.7% vs 20.5%) but it was statistically not significant. Though this study reiterated our findings in terms of survival benefits with local treatment, certain caveats need consideration. Like previous studies in the literature, this study also had a substantial proportion of cN1 disease (43.2%), wherein the radical treatment is the standard of care and hence, this tends to introduce bias in favor of local treatment. Furthermore, they used the threshold of 50Gy as the definition of adequate radiotherapy and excluded patients with single-agent chemotherapy, which implies cautious inference of their results. Another point of contemplation is the utilization of neoadjuvant chemotherapy. The use of neoadjuvant chemotherapy is the standard of care in patients with muscle-invasive bladder cancer. However, the role of neoadjuvant chemotherapy in bladder-sparing chemoradiation regimens for advanced bladder cancer is still evolving. Zapatero et al. showed approximately 80% bladder-sparing rate with neoadjuvant chemotherapy and CRT with comparable overall survival and cancer-specific survival as radical cystectomy in patients with muscle-invasive disease (20). In another recent phase II pilot study, Shi et al. (21) showed a 3-year overall survival of 88% and relapse-free survival of 60% among 59 patients with muscle-invasive and locally advanced bladder cancer (75% having cT3-T4 disease), which was actually superior to radical cystectomy arm. However, an important aspect is the response to neoadjuvant chemotherapy. Only 52% of these patients had a complete response (defined as ≤T1 disease) and proceeded to CRT, compared to those with incomplete response who then underwent radical cystectomy. This potential bias needs to be taken into consideration with the inference of these results and further reiterates the need for accurate biomarkers to predict the response to neoadjuvant chemotherapy for better management of these patients.

Our study has several limitations. Cancer-specific survival could not be studied using the NCDB, as this data is not available. Inaccurate clinical staging is an inherent limitation of retrospective databases including NCDB. As with any retrospective study, despite our attempts to comprehensively address sources of bias, the results may be subject to residual confounding. The NCDB provides limited information on the type and dosing of systemic treatment, which can impact survival. The potential bias due to sub-optimal systemic therapy, specifically in the ST cohort (wherein patients are older with higher comorbidities and some likely being cisplatin-ineligible) could not be addressed. The NCDB also does not provide information on the quality of transurethral resection of bladder tumors before radiation therapy or the variation in concurrent use of chemotherapy with radiation. Quality of life remains an important parameter in managing cancer patients, especially in such locally advanced cancers, which could not be studied using NCDB.

Despite these limitations, our study for the first time systematically compared the three modalities of treatment- RC-PLND, CRT, and CT in patients with stage IIIB bladder cancer using a large cohort from NCDB. Though previous studies have reported comparative analysis for node-positive bladder cancer patients, results were biased due to the inclusion of cN1 patients and non-standardized definitions of CRT. Our study showed the utility of radical treatment in these patients and the equivalence of surgery with CRT in terms of OS.

## 5 Conclusion

In patients with bladder cancer with advanced lymphadenopathy (Stage IIIB), both RC with PLND and concurrent CRT offer equivalent survival benefits and are superior to systemic therapy alone. Both RC+PLND and CRT appeared to be independent predictors for OS.

## Data availability statement

The original contributions presented in the study are included in the article/**Supplementary Material**. Further inquiries can be directed to the corresponding author.

## Ethics statement

Ethical approval was not required for the study involving humans in accordance with the local legislation and institutional requirements. Written informed consent to participate in this study was not required from the participants or the participants’ legal guardians/next of kin in accordance with the national legislation and the institutional requirements.

## Author contributions

Conception and design: HG, AM. Acquisition of data: HG. Analysis and interpretation of data: HG, MB, AM. Drafting of the manuscript: HG, AM. Critical revision of the manuscript for important intellectual content: FD, DK, RS, ML, AM. Statistical analysis: MB. Obtaining funding: AM. Administrative, technical, or material support: AM. Supervision: AM. All authors contributed to the article and approved the submitted version.

## Supplementary material

Supplementary Appendix 1 Definitions of inclusion and exclusion criteria including codes used in National Cancer Database.
