# Supplementary material for: A comparative analysis of radical cystectomy with perioperative chemotherapy, chemoradiation therapy, or systemic therapy in patients with clinically advanced node-positive bladder cancer (cN2/N3)
Source: Front Oncol. 2024 Jan 11;13:1157880. doi: 10.3389/fonc.2023.1157880 (PMC10808589; doi:10.3389/fonc.2023.1157880)
Supplement: Supplementary Appendix 1 — Definitions of inclusion and exclusion criteria including codes used in National Cancer Database. [file DataSheet_1.docx]

**Supplementary Appendix 1**

**Definitions for inclusion and exclusion criteria**

**Histology**

The histologic categories for cases included in both the patient-level analysis of the National

Cancer Database (NCDB) and the interrupted time-series analysis of Surveillance, Epidemiology, and End Results (SEER) data were defined by the following International Classification of Disease for Oncology,

3rd edition, codes:

Urothelial carcinoma:

**Stage**

For the patient-level analysis, we defined stage using the NCDB American Joint Committee on

**Cancer (AJCC) clinical stage variable:**

TNM_CLIN_T

TNM_CLIN_N

TNM_CLIN_M

AJCC_TNM_CLIN_M

AJCC_TNM_CLIN_N

AJCC_TNM_CLIN_T

**Radical cystectomy**

For the patient-level analysis, we defined radical proctectomy using the following NCDB variables

RX_SUMM_SURG_PRIM_SITE codes: 50 and 70.

**Systemic Therapy**

RX_SUMM_CHEMO codes as ‘1,2,3’ for receipt of chemotherapy

**Radiation Therapy Dose**

Variable: TOTAL_DOSE defined the total dose of radiation given for individual patient in Gy

**Supplementary Table 1: Multivariate and Inverse probability weighting**(**IPW)-adjusted Cox regression model predicting overall survival in unweighted and weighted population of stage IIIB bladder cancer**

| **Variable** | **Multivariate regression analysis on unweighted population** | | **IPW-adjusted Cox regression model (weighted Group A and Group B)** | | **IPW-adjusted Cox regression model (weighted Group A and Group C)** | | **IPW-adjusted Cox regression model (weighted Group B and Group C)** | |
| --- | --- | --- | --- | --- | --- | --- | --- | --- |
|  | **Hazard Ratio (95%CI)** | **p-value** | **Hazard Ratio (95%CI)** | **p-value** | **Hazard Ratio (95%CI)** | **p-value** | **Hazard Ratio (95%CI)** | **p-value** |
| **Age,** |  |  |  |  |  |  |  |  |
| <60 | Ref |  | Ref |  | Ref |  | Ref |  |
| 60 - 69 | 0.92 (0.80-1.06) | 0.266 | 0.79 (0.69-0.90) | ***<0.001**** | 0.87 (0.78-0.96) | ***0.007**** | 0.74 (0.64-0.87) | ***<0.001**** |
| 70 - 79 | 1.06 (0.90-1.25) | 0.465 | 0.91 (0.78-1.07) | 0.265 | 0.98 (0.87-1.11) | 0.771 | 0.87 (0.72-1.05) | 0.149 |
| ≥80 | 1.66 (1.36-2.03) | ***0.001**** | 1.49 (1.21-1.83) | ***0.001**** | 1.52 (1.31-1.77) | ***<0.001**** | 1.13 (0.91-1.40) | 0.255 |
| **Gender** |  |  |  |  |  |  |  |  |
| Female | Ref |  | Ref |  | Ref |  | Ref |  |
| Male | 0.83 (0.74-0.92) | ***0.001**** | 0.83 (0.75-0.92) | ***0.001**** | 0.85 (0.79-0.93) | ***<0.001**** | 0.79 (0.71-0.89) | ***<0.001**** |
| **Race,** |  |  |  |  |  |  |  |  |
| White | Ref | 0.744 | Ref |  | Ref |  | Ref |  |
| Black | 0.97 (0.81-1.16) | 0.572 | 1.12 (0.95-1.33) | 0.163 | 0.99 (0.86-1.13) | 0.844 | 0.97 (0.80-1.17) | 0.722 |
| Asian | 0.88 (0.56-1.37) | 0.412 | 0.96 (0.67-1.38) | 0.825 | 0.89 (0.64-1.25) | 0.507 | 1.04 (0.64-1.67) | 0.885 |
| **Ethnicity,** |  |  |  |  |  |  |  |  |
| Non-Hispanic | Ref |  | Ref |  | Ref |  | Ref |  |
| Hispanic | ***0.66 (0.50-0.88)*** | ***0.005**** | 0.47 (0.35-0.63) | ***<0.001**** | 0.69 (0.55-0.85) | ***0.001**** | 0.4 (0.29-0.56) | ***<0.001**** |
| **Charlson Comorbidity Index (CCI)** | | | | | | | | |
| CCI=0 | Ref |  | Ref |  | Ref |  | Ref |  |
| CCI=1 | 1.04 (0.92-1.17) | 0.536 | 1.19 (1.06-1.35) | ***0.003**** | 1.05 (0.95-1.15) | 0.329 | 1.02 (0.89-1.16) | 0.816 |
| CCI=2 | 1.14 (0.94-1.40) | 0.186 | 0.92 (0.75-1.13) | 0.419 | 1.13 (0.98-1.32) | 0.095 | 0.93 (0.75-1.16) | 0.538 |
| CCI=3 | 1.45 (1.11-1.90) | ***0.006**** | 1.35 (1.05-1.74) | ***0.018**** | 1.28 (1.03-1.60) | ***0.025**** | 1.46 (1.15-1.87) | ***0.002**** |
| **Urbanization** |  |  |  |  |  |  |  |  |
| Rural | Ref |  | Ref |  | Ref |  | Ref |  |
| Urban | 0.81 (0.58-1.15) | 0.239 | 1.14 (0.81-1.60) | 0.445 | 0.87 (0.66-1.14) | 0.311 | 0.91 (0.63-1.32) | 0.630 |
| Metro Areas | 0.88 (0.64-1.23) | 0.467 | 1.19 (0.86-1.65) | 0.298 | 0.96 (0.73-1.25) | 0.754 | 0.88 (0.62-1.25) | 0.475 |
| **Insurance Type** |  |  |  |  |  |  |  |  |
| Not Insured | Ref |  | Ref |  | Ref |  | Ref |  |
| Medicare | 1.13 (0.85-1.51) | 0.393 | 1.21 (0.89-1.64) | 0.217 | 1.08 (0.87-1.33) | 0.479 | 1.33 (0.98-1.81) | 0.068 |
| Medicaid | 1.2 (0.87-1.65) | 0.259 | 1.46 (1.05-2.02) | ***0.023**** | 1.01 (0.80-1.28) | 0.936 | 1.49 (1.07-2.07) | ***0.020**** |
| Private | 0.9 (0.68-1.19) | 0.440 | 1 (0.74-1.34) | 0.990 | 0.87 (0.70-1.07) | 0.179 | 1 (0.74-1.35) | 0.998 |
| Other Government | 1.03 (0.64-1.68) | 0.892 | 1.42 (0.88-2.28) | 0.152 | 0.95 (0.65-1.39) | 0.792 | 2 (1.24-3.24) | ***0.005**** |
| **Education** |  |  |  |  |  |  |  |  |
| <7% | Ref |  | Ref |  | Ref |  | Ref |  |
| 7%-12.9% | 1.07 (0.93-1.24) | 0.328 | 1.09 (0.95-1.25) | 0.244 | 1.1 (0.99-1.22) | 0.078 | 0.88 (0.76-1.03) | 0.117 |
| 13-20.9% | 1.07 (0.91-1.26) | 0.405 | 1.08 (0.92-1.26) | 0.360 | 1.05 (0.93-1.18) | 0.464 | 0.83 (0.70-0.99) | ***0.037**** |
| ≥21% | 0.97 (0.79-1.18) | 0.732 | 0.93 (0.78-1.12) | 0.465 | 0.94 (0.81-1.09) | 0.428 | 0.74 (0.60-0.92) | ***0.006**** |
| **Income** |  |  |  |  |  |  |  |  |
| < $38,000 | Ref |  | Ref |  | Ref |  | Ref |  |
| $38,000 - $47,999 | 0.89 (0.76-1.04) | 0.148 | 0.66 (0.57-0.78) | ***<0.001**** | 0.91 (0.81-1.03) | 0.155 | 0.79 (0.66-0.94) | ***0.008**** |
| $48,000 - $62,999 | 0.87 (0.73-1.03) | 0.105 | 0.7 (0.60-0.82) | ***<0.001**** | 0.9 (0.79-1.02) | 0.103 | 0.81 (0.67-0.97) | ***0.021**** |
| ≥$63,000 | 0.79 (0.65-0.96) | ***0.016**** | 0.64 (0.53-0.77) | ***<0.001**** | 0.77 (0.67-0.89) | ***<0.001**** | 0.7 (0.57-0.87) | ***0.001**** |
| **Facility Type** |  |  |  |  |  |  |  |  |
| Community Cancer Program | Ref |  | Ref |  | Ref |  | Ref |  |
| Comprehensive Community Cancer Program | 0.89 (0.72-1.10) | 0.274 | 0.74 (0.59-0.91) | ***0.005**** | 0.9 (0.77-1.06) | 0.223 | 1.06 (0.86-1.31) | 0.568 |
| Academic/Research Program | 0.83 (0.67-1.02) | 0.078 | 0.75 (0.61-0.92) | ***0.006**** | 0.84 (0.72-0.99) | ***0.032**** | 1.02 (0.83-1.26) | 0.830 |
| Integrated Network | 0.92 (0.73-1.15) | 0.455 | 0.7 (0.55-0.88) | ***0.003**** | 0.9 (0.76-1.08) | 0.253 | 1.05 (0.83-1.32) | 0.672 |
| **Preoperative cT stage** | |  |  |  |  |  |  |  |
| cT1 | Ref |  | Ref |  | Ref |  | Ref |  |
| cT2 | 1.08 (0.90-1.31) | 0.409 | 1.1 (0.89-1.37) | 0.362 | 1.25 (1.07-1.46) | ***0.004**** | 0.9 (0.76-1.07) | 0.237 |
| cT3 | 1.4 (1.15-1.71) | ***0.001**** | 1.25 (1.00-1.55) | 0.049 | 1.65 (1.41-1.93) | ***<0.001**** | 0.99 (0.82-1.20) | 0.949 |
| cT4 | 1.68 (1.38-2.06) | ***<0.001**** | 1.78 (1.43-2.23) | ***<0.001**** | 1.92 (1.63-2.25) | ***<0.001**** | 1.41 (1.16-1.70) | ***0.001**** |
| **cN stage** |  |  |  |  |  |  |  |  |
| cN2, n(%) | Ref |  | Ref |  | Ref |  | Ref |  |
| cN3, n(%) | 1.02 (0.90-1.15) | 0.771 | 1.11 (0.98-1.26) | 0.091 | 0.98 (0.89-1.08) | 0.702 | 1.08 (0.97-1.22) | 0.173 |
| **Treatment modality** | |  |  |  |  |  |  |  |
| Systemic therapy | Ref |  |  |  | Ref |  | Ref |  |
| Radical cystectomy | 0.72 (0.65-0.80) | ***<0.001**** | Ref |  | 0.71 (0.66-0.76) | ***<0.001**** | - |  |
| Concurrent CRT | 0.7 (0.59-0.82) | ***<0.001**** | 0.99 (0.91-1.09) | 0.901 | - |  | 0.74 (0.67-0.82) | ***<0.001**** |

CRT- Chemoradiation therapy, **p<0.05 is considered statistically significant*
